# Adaptation of the Care Ecosystem Intervention for Individuals with Dementia in a High-Risk, Care Management Program

**DOI:** 10.1093/geroni/igab046.665

**Published:** 2021-12-17

**Authors:** Brent Forester, Karen Donelan, Christine Vogeli, Christine Ritchie

**Affiliations:** 1 McLean Hospital, McLean Hospital, Massachusetts, United States; 2 Heller School for Social Policy Management, Brandeis University, Waltham, Massachusetts, United States; 3 Mass General Brigham, Somerville, Massachusetts, United States; 4 Massachusetts General Hospital, Boston, Massachusetts, United States

## Abstract

The Care Ecosystem (CareEco) model is a telephone-based dementia care program providing standardized, personalized and scalable support and education for caregivers and persons living with dementia (PLWD), medication guidance, and promotion of proactive decision-making. It has demonstrated improvement in quality of life for PLWD and reduced unnecessary healthcare expenditures. We initiated a pragmatic, embedded randomized pilot trial of an adapted CareEco model for nurses who provide high-risk care management and are embedded in primary care practices within a large healthcare system. Outcomes include feasibility of collecting emergency department visits, usability and acceptability of the intervention by nurse care managers, caregiver strain, behavioral symptoms of dementia and healthcare expenditures. Challenges of implementation include engaging key care management leaders, adaptation of the CareEco training modules for nurses, identification of primary caregivers, training and reinforcing knowledge and skills of the nurses, embedding clinical assessments into care manager workflows and integration with the EMR.

